# Infantile neuroblastoma and maternal occupational exposure to medical agents

**DOI:** 10.1038/s41390-021-01634-z

**Published:** 2021-07-09

**Authors:** Yuhki Koga, Masafumi Sanefuji, Syunichiro Toya, Utako Oba, Kentaro Nakashima, Hiroaki Ono, Shunsuke Yamamoto, Maya Suzuki, Yuri Sonoda, Masanobu Ogawa, Hiroyuki Yamamoto, Koichi Kusuhara, Shouichi Ohga, The Japan Environment and Children’s Study (JECS) Group, Michihiro Kamijima, Shin Yamazaki, Yukihiro Ohya, Reiko Kishi, Nobuo Yaegashi, Koichi Hashimoto, Chisato Mori, Shuichi Ito, Zentaro Yamagata, Hidekuni Inadera, Takeo Nakayama, Hiroyasu Iso, Masayuki Shima, Youichi Kurozawa, Narufumi Suganuma, Koichi Kusuhara, Takahiko Katoh

**Affiliations:** 1 Department of Pediatrics, Graduate School of Medical Sciences, Kyushu University, Fukuoka, Japan; 2 Research Center for Environment and Developmental Medical Sciences, Kyushu University, Fukuoka, Japan; 3 Department of Pediatrics, University of Occupational and Environmental Health, Kitakyushu, Japan; 4 Regional Center for Japan Environment and Children’s Study, University of Occupational and Environmental Health, Kitakyushu, Japan; 5 Principal investigator, Nagoya City University, Nagoya, Japan; 6 National Institute for Environmental Studies, Tsukuba, Japan; 7 National Center for Child Health and Development, Tokyo, Japan; 8 Hokkaido University, Sapporo, Japan; 9 Tohoku University, Sendai, Japan; 10 Fukushima Medical University, Fukushima, Japan; 11 Chiba University, Chiba, Japan; 12 Yokohama City University, Yokohama, Japan; 13 University of Yamanashi, Chuo, Japan; 14 University of Toyama, Toyama, Japan; 15 Kyoto University, Kyoto, Japan; 16 Osaka University, Suita, Japan; 17 Hyogo College of Medicine, Nishinomiya, Japan; 18 Tottori University, Yonago, Japan; 19 Kochi University, Nankoku, Japan; 20 University of Occupational and Environmental Health, Kitakyushu, Japan; 21 Kumamoto University, Kumamoto, Japan

## Abstract

**Background:**

Healthcare workers are often exposed to hazardous agents and are at risk for adverse health consequences that affect not only themselves but also their infants. This study aimed to examine whether such occupational exposure increased the risk of childhood cancer in offspring.

**Methods:**

We used the dataset of the Japan Environment and Children’s Study, a nationwide birth cohort involving over 100,000 mother–child pairs. Information was obtained via successive questionnaires that were completed until the child turned 1 year of age. The parents were asked whether they occupationally handled medical agents during pregnancy.

**Results:**

A total of 26 infants developed neoplasms: neuroblastoma, leukemia, and brain tumor. The incidence of neuroblastoma was significantly higher in infants whose mothers were exposed to radiation (3/2142: 140.1 per 100,000 population) than in those who were not (12/90,384: 13.3 per 100,000 population). Multivariable regression analyses revealed a close association between maternal irradiation and the development of neuroblastoma (adjusted incident rate ratio: 10.68 [95% confidence interval: 2.98‒38.27]).

**Conclusions:**

The present study demonstrated, for the first time, a potential association between maternal occupational exposure and the occurrence of neuroblastoma in offspring. Further studies involving the large pediatric cancer registries are needed to confirm these preliminary results.

**Impact:**

- Healthcare workers are often exposed to hazardous agents and are at risk for adverse health consequences that affect not only themselves but also their infants.
- This study examined the association between such occupational exposure and offspring’s cancers that developed until the age of 1 year.
- Maternal exposure to ionizing radiation was associated with infantile neuroblastoma in offspring.
- Further studies involving the large pediatric cancer registries are needed to confirm these preliminary results.

## Introduction

Cancer is rare in childhood but a severe life-threatening disease. Although the etiology of childhood cancer remains uncertain, several studies have suggested that parental exposure to various environmental factors plays a role in the development of cancers in offspring. A large number of studies, which almost always used a case–control design, have reported that the environmental factors are suspected to be air pollution, pesticides, and cigarette smoke in the living environment of the parents.^1^ In addition, some parents may be occupationally exposed to specific environment that has a potential carcinogenic effect on childhood cancer.^2^ Among various occupations, healthcare workers often handle hazardous agents, such as ionizing radiation and anticancer drugs in their workplaces. It has previously been demonstrated that ionizing radiation promotes the development of childhood leukemia.^3^ Anticancer drugs increase the risk of acute leukemia in the healthcare workers who handle them.^4^ However, to our knowledge, no studies have comprehensively addressed the relationship between occupational exposure to such medical agents and infantile cancer in offspring.

The goal of the current study was to investigate the association between parental occupational exposure to medical agents and pediatric malignancy in children of ≤1 year of age using data from the Japan Environment and Children’s Study (JECS). We examined neuroblastoma, leukemia, and brain tumor because these are the most frequent cancers that occur during the first year of life in Japan.^5^ Former studies have demonstrated the possible association of intrauterine exposure to ionizing radiation with leukemia^6^ but not with neuroblastoma^7,8^ and brain tumor.^9,10^ However, information is still lacking on such association, especially for neuroblastoma and brain tumor. This nationwide prospective birth cohort includes over 100,000 children with a sufficiently analyzable number of patients with neoplasms. Exploring the influence of such parental exposure on life-threatening diseases in offspring is of great importance to provide a preliminary finding to review and control the occupational environments in which pregnant women and their partners work.

## Methods

### Study design

The JECS is a nationwide, multicenter, prospective birth cohort study, funded by the Ministry of Environment, Japan, and the details of the study design have been described elsewhere.^11,12^ Briefly, pregnant participants and their partners were registered between January 2011 and March 2014. During pregnancy, information including demographics and occupational exposure was obtained twice during the first and second/third trimesters from the pregnant women and once from their partner, using self-administered questionnaires. Detailed information regarding the mother and child was obtained from medical records during the first trimester, at the time of delivery, and when the child was 1 month of age. After delivery, information (including diseases affecting the child) was collected at 1 month and 6 months of age, and every 6 months until 6 years of child’s age, and twice a year thereafter via self-reported questionnaires that were completed by the parents. The JECS protocol was reviewed and approved by the Ministry of Environment’s Institutional Review Board for Epidemiological Studies (#100910001) and by the ethics committees of all the participating institutions (#2019-070). Ethical approval for this study was an extension of the ethical approval for the JECS protocol. The JECS was conducted in accordance with the Declaration of Helsinki. Written informed consent was obtained from all parents.

### Participants

**Fig. 1:**
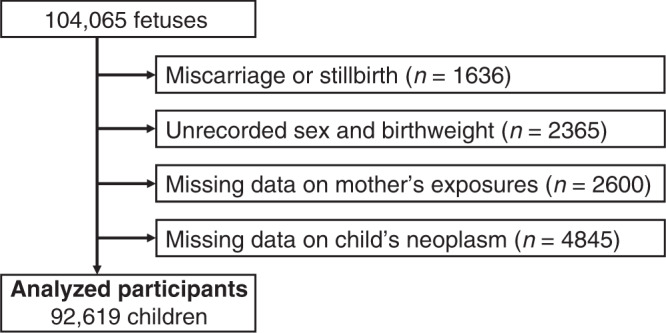
Flowchart of participant selection.

In this study, we used the “jecs-an-20180131” fixed dataset, which was released in March 2018. The dataset contains all of the available data extracted from the above questionnaires and records until the child reached 12 months of age. The data for 104,065 fetuses from 103,062 pregnancies were linked to respective maternal data. The data were also linked to the respective paternal data when their fathers were registered (*n* = 52,415). Among the 104,065 fetuses, we excluded 1636 children who resulted in miscarriage or stillbirth, and further 2365 children who had missing information on sex or birthweight. We additionally excluded 7445 children who had missing information on the mother’s exposure to medical agents during pregnancy, or the diagnosis of the child’s neoplasms within 1 year of age. After these exclusions, data for 92,619 children were included in our analysis (Fig. 1).

### Exposure and outcome

Parental occupational exposure to three medical agents, ionizing radiation, anticancer drugs, and anesthetics, was determined using questionnaires during pregnancy.^13^ Ionizing radiation included radioactive rays, materials, and isotopes. Parents were asked whether they handled these agents for over half a day at work for reasons other than their own treatment. The exposure periods were set as “during pregnancy” for mothers and “three months before awareness of partner’s pregnancy,” which corresponds to the preconception period, for fathers. They were requested to fill in the frequency with one of the following choices: less than once a month, once to three times a month, once to six times a week, and every day. A parent was defined as “exposed” if they handled medical agents once a month or more frequently in either of the questionnaires at the first or second/third trimester for mothers or in the questionnaire for fathers. The outcome was the development of neoplasms by 1 year of age. Parents were asked whether their child was diagnosed with neuroblastoma, leukemia, or brain tumor by physicians, via questionnaires at either 6 or 12 months of age.

### Statistical analysis

To obtain an overview of the relationship between parental exposure and neoplasms in offspring, we produced cross-tabulation tables and performed Fisher’s exact tests as a statistical analysis. We also conducted a stepwise regression analysis adjusted for the following covariates: birthweight, paternal and maternal education levels (junior high school; high school; university or graduate school), annual family income (low: <4,000,000; middle: 4,000,000–5,999,999; high: ≥6,000,000 Japanese yen). Maternal alcohol consumption is suspected to increase the risk of neuroblastoma,^14^ but was not included in this regression model because it was not consumed during pregnancy in any mothers of children with subsequent neuroblastoma. Statistical analyses were performed using the R software program (version 4.0.3). A Poisson regression model using robust standard errors was employed to estimate the adjusted incident rate ratio and 95% confidence interval, using “sandwich” version 3.3-0 in the R package.

## Results

The baseline characteristics of the 92,619 children are shown in Table 1. During pregnancy, ionizing radiation, anticancer drugs, and anesthetics were handled occupationally by 2142 (2.3%), 1298 (1.4%), and 1015 (1.1%) mothers, respectively. By 1 year of age, 26 children developed the following neoplasms: neuroblastoma (*n* = 15), leukemia (*n* = 8), and brain tumor (*n* = 3, Table 2). Their mothers had no medical history of malignancies, hypertension, diabetes mellitus, hyperthyroidism, hypothyroidism, psychiatric diseases, epilepsy, kidney diseases, and autoimmune diseases. Of the 2142 children whose mothers were exposed to radiation, three developed neuroblastoma. Their mothers were very unlikely to receive medical exposure to radiation and/or anticancer drugs for their own treatment because they had no history of malignancies. The incidence rate (140.1 per 100,000 population) was significantly higher than that (13.3 per 100,000) of the children without maternal exposure (*p* = 0.005). The agents and timing of the exposure in utero were different among the three children: one was exposed to radiation and anticancer drugs during the first and second/third trimester and anesthetics during the first trimester; another to all the three agents during the second/third trimester; the other to irradiation alone during the second/third trimester. Conversely, children who were exposed to anticancer drugs or anesthetics but not radiation did not develop subsequent neuroblastoma (data not shown), suggesting that radiation was a requisite for the development of neuroblastoma. Leukemia and brain tumors did not occur in children whose mothers were exposed to any of the three agents. A multivariable-adjusted regression analysis confirmed the association between maternal exposure to ionizing radiation and neuroblastoma in offspring (incident rate ratio: 10.68 [95% confidence interval: 2.98‒38.27] adjusted for paternal education level after stepwise selection).

**Table 1:** Baseline characteristics of the children (*n* = 92,619).

		[Missing]
Male	47,426 (51.2%)	0
Gestational age (week) (mean ± SD)	39.2 ± 1.6	0
Birth weight (g) (mean ± SD)	3013 ± 427	0
Maternal age (years) (mean ± SD)	31.3 ± 5.0	4
Maternal education		840
Junior high school	3990 (4.3%)	
High school	67,418 (73.5%)	
University/graduate school	20,371 (22.2%)	
Paternal education		1359
Junior high school	6278 (6.9%)	
High school	53,975 (59.1%)	
University/graduate school	31,007 (34.0%)	
Family income		6631
Low (<4,000,000 JPY)	34,034 (39.6%)	
Middle (4,000,000–5,999,999 JPY)	28,627 (33.3%)	
High (≥6,000,000 JPY)	23,327 (26.1%)	
Maternal alcohol during pregnancy	2511 (2.7%)	1157
Maternal exposure during pregnancy		
Ionizing radiation	2142 (2.3%)	93
Anticancer drugs	1298 (1.4%)	70
Anesthetics	1015 (1.1%)	57
Paternal exposure during preconception		
Ionizing radiation	1446 (3.2%)	47,848
Anticancer drugs	289 (0.6%)	47,582
Anesthetics	328 (0.7%)	47,568

*JPY* Japanese yen, *SD* standard deviation.

**Table 2:** Association between parents’ exposure to ionizing radiation and child’s neoplasms.

		Neuroblastoma^a^	Leukemia	Brain tumor
Maternal exposure		Number (/100k)	Number (/100k)	Number (/100k)
No	90,384 (97.7%)	12 (13.3)	8 (8.9)	3 (3.3)
Yes	2142 (2.3%)	3^b^ (140.1)	0 (0.0)	0 (0.0)
		Neuroblastoma	Leukemia	Brain tumor
Paternal exposure		Number (/100k)	Number (/100k)	Number (/100k)
No	43,325 (96.8%)	7 (16.2)	3 (6.9)	3 (6.9)
Yes	1446 (3.2%)	0 (0)	0 (0.0)	0 (0.0)

*/100k* per 100,000 children.

^a^*p* = 0.005.

^b^Two were concurrently exposed to both anticancer and anesthetic drugs.

Information on paternal exposure was available for almost half of the participants (Table 1). During the preconception period, ionizing radiation, anticancer drugs, and anesthetics were handled occupationally by 1446 (3.2%), 289 (0.6%), and 328 (0.7%) fathers, respectively. Among them, a total of 13 children developed the following neoplasms: neuroblastoma (*n* = 7), leukemia (*n* = 3), and brain tumor (*n* = 3, Table 2). No associations were observed between paternal exposure and neoplasms in offspring.

## Discussion

The current study investigated the relationship between parental occupational exposure to ionizing radiation, anticancer drugs, and anesthetics during pregnancy and neoplasms in children who developed by 1 year of age. The incidence of neuroblastoma in offspring was higher among children of mothers who handled these agents than those who did not. The result was confirmed in a multivariable regression analysis with adjustment for confounders. The results regarding leukemia and brain tumors were inconclusive because of the small numbers of these patients. Our study demonstrated, for the first time, a potential association between maternal exposure to some medical agents at work and infantile neuroblastoma in offspring.

This is one of the largest prospective birth cohort studies to examine the association between parental occupational exposure and childhood cancer. Considering that almost all of the previous studies used a case–control study design, this prospective cohort, representing the Japanese general population, yielded less selection bias than the case–control studies. The big cohort included a sufficient number of children with neuroblastoma by 1 year of age, although the numbers of patients with leukemia and brain tumors were very small during the observation period. The exposure factors were also prospectively collected before the onset of these cancers, eliminating the risk of recall bias.

The association between neuroblastoma and various environmental factors has been reported but remains inconclusive. Neuroblastoma, a sympathetic nervous system tumor originating from the neural crest, is the most common tumor in children of <1 year of age. The steep decrease in the incidence of the disease after infancy suggests the importance of the in utero environment to its development. Although a small portion of cases has genetic predisposition, the majority of cases are sporadic and the etiology remains poorly understood.^14^ Several studies have reported an association between neuroblastoma and parental occupational exposure to various factors during periods from preconception to birth, including electromagnetic fields,^15–17^ pesticides,^18^ and chemicals;^19,20^ other studies showed controversial findings.^21–23^ To the best of our knowledge, only a few studies demonstrated a null association between parental exposure to medical ionizing radiation and neuroblastoma in offspring,^7,8^ and no previous studies have examined the association with anticancer drugs or anesthetics.

The present results seem well explained by previously reported evidence to support that childhood cancer can be caused by intrauterine—but not preconception—exposure to ionizing radiation.^24^ Ionizing radiation has the potential to produce DNA damage and thus promote carcinogenesis. In our study, the exposure periods were set as during pregnancy for mothers and the preconception period for fathers. Although a case–control study found a strong association between preconception radiation exposure in the fathers of children with hematological malignancies,^25^ subsequent studies have argued against such human germ-cell mutagenesis of ionizing radiation in nuclear installation workers^26–32^ and atomic bomb survivors,^33^ based mainly on the absence of a dose–response relationship.^34^ Conversely, several former studies have demonstrated the association between maternal exposure from radiographic examinations during pregnancy and overall childhood cancer,^35–37^ especially with leukemia,^6^ whereas later studies reported negligible results,^9^ probably because of the declining doses of radiation over the decades.^38^ Furthermore, a few studies reported that the incidence of childhood cancer was significantly high in female radiation workers; however, the overall number of cases was small.^26,29,30^ The positive association of offspring’s neuroblastoma with maternal occupational radiation in our study is in contrast to the null association with maternal medical radiation in former studies.^7,8^ This discrepancy might possibly be due to frequent occupational exposures to minimal levels of ionizing radiation compared with occasional medical exposures to a bit higher levels.

In contrast to ionizing radiation, there is little evidence regarding the effects of occupational exposure to anticancer drugs on childhood cancer. Healthcare workers, including nurses and pharmacists, are exposed to anticancer drugs and are at high risk for adverse health effects.^39–41^ Cumulative exposure to these drugs at low doses over years can induce genetic damage,^42^ potentially leading to carcinogenesis, teratogenesis, and adverse reproductive effects in the workers who are exposed to them.^43–46^ To our knowledge, no studies have addressed the effects of parental exposure to anticancer drugs on illnesses in offspring. Although transplacental exposure in offspring is much shorter than parental occupational exposure, these agents may have a more profound impact on the developing fetus than they do on working adults. Maternal anesthetic exposure appeared to have a little causal relationship with neuroblastoma in offspring; however, further investigation is needed.

The present study was associated with some limitations. First, the exact process, timing, duration, and dose of exposure, as well as the subtypes of neoplasms, were not determined because the information was obtained from self-administered questionnaires. Second, the number of infants with neuroblastoma was relatively small, even in this large dataset. The small number yields the wide confidence intervals of the estimated incident rate ratio, which may be imprecise. It also precluded not only examining the risk of neuroblastoma with respect to frequency of exposure but also including many confounding factors to be adjusted for in regression analyses without stepwise selection. Furthermore, the effects of paternal exposure were more inconclusive because data of paternal exposure were not obtained for almost half of the fathers. Finally, the present study cannot determine whether radiation alone or in combination with other agents was really associated with childhood neuroblastoma because these medical agents were often handled concurrently. The cumulative incidence of neoplasms would be expected to increase as the children registered in JECS become older, probably enabling the elucidation of differential effects among these agents.

In conclusion, the present study provided the preliminary findings of a potential association between maternal occupational exposure to medical agents and neuroblastoma in offspring. To verify this association, the elucidation of biological mechanisms using an animal model and a larger detailed investigation involving an international childhood cancer registry are needed.
